# Inspiratory muscle training to facilitate weaning from mechanical ventilation: protocol for a systematic review

**DOI:** 10.1186/1756-0500-4-283

**Published:** 2011-08-11

**Authors:** Lisa H Moodie, Julie C Reeve, Niki Vermeulen, Mark R Elkins

**Affiliations:** 1 Physiotherapy Department, Greenlane Clinical Centre, Auckland, New Zealand; 2 Division of Rehabilitation and Occupation Studies, Faculty of Health and Environmental Studies, AUT University, Auckland, New Zealand; 3 Department of Social Studies of Science, University of Vienna, Austria; 4 Department of Respiratory Medicine, Royal Prince Alfred Hospital, Sydney, Australia

## Abstract

**Background:**

In intensive care, weaning is the term used for the process of withdrawal of mechanical ventilation to enable spontaneous breathing to be re-established. Inspiratory muscle weakness and deconditioning are common in patients receiving mechanical ventilation, especially that of prolonged duration. Inspiratory muscle training could limit or reverse these unhelpful sequelae and facilitate more rapid and successful weaning.

**Methods:**

This review will involve systematic searching of five electronic databases to allow the identification of randomised trials of inspiratory muscle training in intubated and ventilated patients. From these trials, we will extract available data for a list of pre-defined outcomes, including maximal inspiratory pressure, the duration of the weaning period, and hospital length of stay. We will also meta-analyse comparable results where possible, and report a summary of the available pool of evidence.

**Discussion:**

The data generated by this review will be the most comprehensive answer available to the question of whether inspiratory muscle training is clinically useful in intensive care. As well as informing clinicians in the intensive care setting, it will also inform healthcare managers deciding whether health professionals with skills in respiratory therapy should be made available to provide this sort of intervention. Through the publication of this protocol, readers will ultimately be able to assess whether the review was conducted according to a pre-defined plan. Researchers will be aware that the review is underway, thereby avoid duplication, and be able to use it as a basis for planning similar reviews.

## Introduction

Mechanical ventilation temporarily replaces or supports spontaneous breathing in patients with critical illness or requiring post-operative support in an intensive care unit (ICU). Weaning is the term used for the process of withdrawal of mechanical ventilation to enable spontaneous breathing to be re-established. Patients are considered to be successfully weaned from ventilatory support when they can breathe on their own for at least 48 hours [1]. Almost 70% of ICU patients proceed from initiation of weaning to successful extubation without difficulty on the first attempt [2]. Other patients have a more difficult or prolonged period of weaning, which is associated with a poorer prognosis [3,4]. The weaning process typically comprises 40 to 50% of the total duration of mechanical ventilation [2]. Despite representing only a small percentage of ICU patients, those who fail to wean from ventilation consume a disproportionate share of resources [1].

Weaning failure resulting in prolonged ventilation is detrimental to the individual as it is associated with increased risk of respiratory muscle weakness, critical illness myopathy/polyneuropathy (CIM/CIP), nosocomial infection and airway trauma [2,5]. Prolonged ventilation is also associated with an increase in mortality, morbidity and ICU length of stay, as well as reduced functional status and quality of life [5-7]. In addition prolonged ventilation is expensive, consuming a large fraction of hospital resources, with a healthcare burden that may continue after hospital discharge [8].

Weakness or fatigue of the diaphragm and accessory muscles of inspiration is widely recognised as a cause of failure to wean from mechanical ventilation [6,9]. Fatigue may be due to excessive load on the inspiratory muscles, which may result from increased airway resistance and/or reduced lung compliance. A reduction in the capacity of the respiratory muscle pump may also occur due to phrenic nerve injury, CIM/CIP, corticosteroids, endocrine or nutritional factors [7]. There is increasing evidence to show mechanical ventilation itself may adversely affect the diaphragm's structure and function, which has been termed ventilator-induced diaphragmatic dysfunction [9]. The combination of positive pressure ventilation and positive end-expiratory pressure may unload the diaphragm therefore subjecting it to changes in myofibre length, which may account for its rapid atrophy [9]. In addition, patients who undergo prolonged periods of ventilation demonstrate a decrease in respiratory muscle endurance and are at risk of respiratory muscle fatigue [10].

Inspiratory muscle training (IMT) is a technique that targets the muscles of inspiration-namely the diaphragm and accessory inspiratory muscles-with the aim of increasing inspiratory muscle strength and endurance. In ventilated patients, IMT can be undertaken in several ways: isocapnic/normocapnic hyperpnoea training, resistive flow training, threshold pressure training, or adjustment of the ventilator to provide a training load for the inspiratory muscles.

### Isocapnic/normocapnic hyperpnoea

Belman [11] first reported training the respiratory muscles using isocapnic hyperpnoea to increase inspiratory muscle endurance and facilitate weaning. This is a method of respiratory muscle endurance training, during spontaneous breathing or mechanical ventilation, in which the patient voluntarily breathes at high levels of ventilation for a sustained period of time, generating a low-pressure high-flow load. This would normally result in hypocapnia but normal PaCO_2 _levels are maintained by entraining CO_2 _into the inspiratory limb of the ventilator circuit. Normocapnic hyperpnoea is not commonly used in clinical practice due to the complexity of the equipment required to maintain CO_2 _homeostasis [12].

### Inspiratory resistive flow training

Abelson and Brewer [13] and Aldrich et al [14] first reported using inspiratory resistive training to attempt to increase inspiratory muscle strength and facilitate weaning from mechanical ventilation. This method involves attaching the IMT device via a connector or adaptor to the endotracheal or tracheostomy tube [13]. This makes the patient inhale through an orifice with a reduced diameter, which in turn places an increased load on the inspiratory muscles. The amount of inspiratory resistance is dependent on the flow generated by the patient, which may be variable if the breathing pattern is not regulated.

### Inspiratory threshold pressure training

In threshold pressure training, a specific negative threshold pressure has to be reached before a spring-loaded valve opens to allow inspiratory flow. The pressure is not influenced by patients modifying their breathing pattern. The IMT device is incorporated into the ventilator circuit with an adaptor or connector. Case reports have described the use of inspiratory pressure threshold training in an attempt to increase inspiratory muscle strength in ventilated patients which was followed by increases in the duration of periods of unassisted breathing [1,15,16].

### Adjustment of ventilator sensitivity

It is possible to alter the ventilator sensitivity to provide resistance and hence a pressure load to the inspiratory muscles. By progressively adjusting the pressure trigger sensitivity, the inspiratory load can be gradually increased. This is typically based on a percentage of the maximal inspiratory pressure (MIP) [17].

Inspiratory muscles respond to training in the same way as other skeletal muscles in regard to the principles of overload, specificity and reversibility [18]. In healthy people and in people with chronic obstructive pulmonary disease, IMT increases respiratory muscle strength and endurance [19-22]. The effectiveness of IMT in increasing inspiratory muscle strength and endurance in ventilated patients (to potentially reduce weaning duration) has not yet been established. In patients who have failed to wean from mechanical ventilation using standard weaning techniques, several case reports have demonstrated increases in inspiratory muscle strength measured by maximal inspiratory pressure (MIP) after IMT, followed by successful weaning [1,13,14]. Sprague et al [1] hypothesised that IMT may work to assist patients in weaning from ventilation by any of the following mechanisms:

1. improving the function of the respiratory muscle pump via changes in muscle fibre type, size and physiological efficiency,

2. improving the activation of the respiratory muscle pump via the adaptation of neural pathways to allow more efficient motor unit recruitment, and

3. improving the breathing pattern.

Improving the strength and endurance of the inspiratory muscles may therefore reduce ventilator dependence over time and facilitate spontaneous breathing. Reducing ventilation time may help to reduce the incidence of ventilator-associated complications and may decrease ICU and hospital length of stay.

To date there are no systematic reviews evaluating the effectiveness of IMT in facilitating weaning from mechanical ventilation. By increasing inspiratory muscle strength and endurance, IMT may have the potential to reduce the duration of mechanical ventilation and so decrease associated complications and costs and improve patient outcomes.

One aim of this systematic review is to evaluate the effectiveness of IMT in increasing inspiratory muscle strength and endurance in mechanically ventilated patients. A further aim is to determine whether IMT affects the duration of weaning from mechanical ventilation, the duration of unassisted breathing periods during the weaning period, and the rate of reintubation.

## Methods

### Inclusion criteria for studies in the review

#### Types of studies

Eligible studies will be randomised controlled trials and quasi-randomised controlled trials. Only the first arm of cross-over trials will be included.

#### Types of participants

Eligible participants will be adults (16 years and over) who are intubated or tracheostomised and are receiving full or partial mechanical ventilation.

#### Types of interventions

Inspiratory muscle training including isocapnic/normocapnic hyperpnoea, inspiratory resistive flow training (e.g. with *Pflex *brand), threshold pressure loading (e.g. with *Threshold *brand), or adjustment of the ventilator sensitivity, compared with sham or no IMT.

#### Types of outcome measures

##### Primary Outcomes

1. Measures of inspiratory muscle strength, such as maximal inspiratory pressure (MIP), if lung volume during the measurement of MIP is controlled

2. Measures of inspiratory muscle endurance, such as sustained MIP or MIP load

3. Duration of unassisted breathing periods after commencement of IMT

4. Weaning duration from the identification of readiness to wean (as determined by the authors and/or commencement of IMT) to the discontinuation of mechanical ventilation

5. Weaning success (proportion of patients successfully weaned, defined as spontaneous breathing without mechanical support for at least 48 hours)

6. Reintubation (proportion of extubated patients who were reintubated within the follow-up period of the study)

##### Secondary outcomes

1. Tracheostomy (proportion of patients tracheostomised) after commencement of IMT or no/sham IMT

2. ICU length of stay

3. Hospital length of stay

4. Mortality

5. Adverse events

### Search Strategy

The following databases will be electronically searched for all available years: PEDro, CENTRAL, PubMed, EMBASE and CINAHL. The search will not be limited by date, language or publication status. We will check the reference lists of any eligible studies identified for further relevant studies. We will also ask authors of eligible trials and manufacturers of IMT devices if they know of other eligible studies. Translation of foreign language trials will be performed by the authors for Dutch and German (NV) and Italian (ME), or by commercial translators for other languages.

### PEDro Search Strategy

1. inspirat* musc* train* in Abstract & Title field

2. respirat* musc* train* in Abstract & Title field

3. respirat* musc* condit* in Abstract & Title field

4. resist* load* in Abstract & Title field

5. press* thresh* load* in Abstract & Title field

6. incre* thresh* load* in Abstract & Title field

### CENTRAL Search Strategy

1. ((muscu* OR muscl*) NEAR/15 (train* OR condition*) NEAR/15 (inspirat* OR ventilat* OR respirat* OR pulmonary)):ti, ab, kw in Clinical Trials

2. ((isocapn* OR normocapn*) NEAR/5 (hyperpn*)):ti, ab, kw in Clinical Trials

3. ((inspirat*) NEAR/5 (resist*) NEAR/5 (load*)):ti, ab, kw in Clinical Trials

4. ((pressure) NEAR/5 (threshold) NEAR/5 (load*)):ti, ab, kw in Clinical Trials

5. (p-flex):ti, ab, kw in Clinical Trials

6. (#1 OR #2 OR #3 OR #4 OR #5)

7. "intensive care":ti, ab, kw in Clinical Trials

8. ((critical*) AND (care OR ill*)):ti, ab, kw in Clinical Trials

9. (intubat* OR ventilat* OR tracheostom*):ti, ab, kw in Clinical Trials

10. (#7 OR #8 OR #9)

11. (#6 AND #10)

### PubMed Search Strategy

1. randomized controlled trial[Publication Type]

2. controlled clinical trial[Publication Type]

3. randomi*ed[Title/Abstract]

4. placebo[Title/Abstract]

5. "clinical trials as topic"[MeSH Major Topic]

6. randomly[Title/Abstract]

7. trial[Title]

8. #1 OR #2 OR #3 OR #4 OR #5 OR #6 OR #7

9. (muscu* OR muscl*) AND (train* OR condition*) AND (inspirat* OR respirat* OR ventilat* OR pulmonary)

10. (isocapn* OR normocapn*) AND (hyperpn*)

11. (inspirat* AND resist* AND load*)

12. (pressure AND threshold AND load*)

13. p-flex

14. #9 OR #10 OR #12 OR #13

15. "intensive care"[Title/Abstract]

16. (critical*[Title/Abstract] AND (care[Title/Abstract] OR ill[Title/Abstract] OR illness[Title/Abstract]))

17. intubat*[Title/Abstract] OR ventilat*[Title/Abstract] OR tracheostomi*[Title/Abstract]

18. #15 OR #16 OR #17

22. #8 AND #14 AND #18

### EMBASE Search Strategy

1. 'intensive care'/exp

2. 'critically ill'/exp

3. 'artificial ventilation'/exp

4. #1 OR #2 OR #3

5. 'breathing exercise'/exp

6. 'breathing muscle'/exp

7. #5 OR #6

8. #4 AND #7

9. #8 AND ([controlled clinical trial]/lim OR [randomized controlled trial]/lim) AND [humans]/lim

### CINAHL Search Strategy

1. randomized controlled trial [Publication Type]

2. controlled clinical trial [Publication Type]

3. clinical trials [Exact Major Subject Heading]

4. random$ [Title]

5. randomly [Abstract]

6. trial [Title]

7. #1 OR #2 OR #3 OR #4 OR #5 OR #6

8. (muscu* OR muscl*) AND (train* OR condition*) AND (inspirat* OR respirat* OR ventilat* OR pulmonary) [Abstract]

9. (isocapn* OR normocapn*) AND (hyperpn*) [Abstract]

10. (inspirat* AND resist* AND load*) [Abstract]

11. (pressure AND threshold AND load*) [Abstract]

12. p-flex [Abstract]

14. #8 OR #9 OR #10 OR #11 OR #12

15. intensive care [Exact Major Subject Heading]

16. #7 AND #14 AND #15

Two authors will independently review all potential studies for inclusion against the eligibility criteria. They will examine the title and abstract and, where necessary, the full text of studies to assess if they are eligible for inclusion. If they cannot reach agreement by discussion, a third author will make the final decision re eligibility.

### Data extraction

Two authors will independently use a standard form to extract study characteristics and outcome data from the studies. Discrepancies will be checked against the original data. A third author will make the final decision if there is a disagreement. LM will enter data in Revman meta-analysis software [23]. We will report data during intubation, at extubation from mechanical ventilation, within 2 days of extubation, at discharge from ICU, more than 2 days since extubation but before discharge from hospital, and at discharge from hospital. Any outcomes measured after discharge from hospital will be grouped as less than 1 month, 1 to 6 months, and over 6 months.

### Quality assessment

Methodological quality will be assessed using the PEDro scale [24,25] by a trained assessor (ME). Scores will be based on all information available from both the published version and the authors themselves. No trial will be excluded on the basis of poor quality.

### Data analysis

For binary (dichotomous) outcome measures, we aim to calculate a pooled estimate of treatment effect for each outcome across studies using risk ratio where appropriate and the corresponding 95% confidence intervals (CIs). For continuous outcome measures, we will calculate a pooled estimate of treatment effect by calculating the mean difference and the corresponding 95% CIs. When analysing count data, a decision will be made whether to treat these as dichotomous, continuous, time-to-an-event or as a rate depending on whichever of these methods allows the greatest number of data points to be included in the meta-analysis. We plan to analyse time-to-event data using the hazard ratio and 95% CIs. When conducting a meta-analysis combining results from crossover studies, we plan to use the first-arm data only. In the event of missing, incomplete, or unclear data we plan to contact the original investigators. If we do not obtain the necessary data for analysis, we will describe the study results in the text.

We plan to assess the degree of heterogeneity between studies using the I^2 ^statistic [26]. This measure describes the percentage of total variation across studies that are caused by heterogeneity rather than by chance [26]. The values of I^2 ^lie between 0% and 100%, and a simplified categorisation of heterogeneity that we plan to use is low (I^2 ^value of less than 25%), moderate (I^2 ^value of between 25 and 50%), and high (I^2 ^value of over 50%) [26]. If sufficient studies are included we will assess reporting bias among the studies using the funnel plot method discussed in the Cochrane Handbook for Systematic Reviews of Interventions [26]. If asymmetry is present, we will explore possible causes including publication bias, methodological quality, and true heterogeneity. We will enter data extracted from included studies into RevMan software [23]. If there is no significant heterogeneity, we will compute pooled estimates of the treatment effect for each outcome under a fixed-effect model. If there is significant heterogeneity, we will compute pooled estimates of the treatment effect for each outcome using a random-effects model.

If there is significant heterogeneity (over 50%) and there are sufficient studies included in the review, we will investigate the possible causes further by performing the following subgroup analyses:

Isocapnic/normocapnic hyperpnoea

Inspiratory resistive flow training

Inspiratory threshold pressure training

Adjustment of ventilator sensitivity

Strength regimens

Endurance regimens

Anticipated to fail weaning prior to commencement of IMT, according to any objective criteria used by authors

Ventilator dependent (having failed at least one previous weaning attempt) prior to commencement of IMT

Duration of ventilation prior to starting IMT

### Sensitivity analysis

We will test the robustness of our results through sensitivity analyses excluding unpublished studies, small studies, and studies with a PEDro score less than 5.

## Discussion and Conclusion

This review aims to provide comprehensive evidence of the effectiveness of IMT at improving inspiratory muscle strength and facilitating weaning in patients who are intubated and mechanically ventilated. This will inform physiotherapists, respiratory therapists and other clinicians in the intensive care setting. It will also inform policy makers and healthcare commissioners in deciding whether health professionals with skills in respiratory therapy should be made available to provide this sort of intervention. Through the publication of this protocol, readers will be able to assess whether the review was conducted according to a pre-defined plan. Researchers will be aware that the review is underway, and thereby avoid duplication. Researchers will also be able to use it as a basis for planning similar reviews.

## Abbreviations

CI: confidence interval; CIM/CIP: critical illness myopathy/critical illness polyneuropathy; ICU: intensive care unit; IMT: inspiratory muscle training; MIP: maximal inspiratory pressure.

## Competing interests

The authors declare that they have no competing interests.

## Authors' contributions

LM wrote the first draft of the protocol. ME drafted the search strategies. All authors contributed to revision of the protocol. All authors read and approved the final manuscript.
